# Different Aspects of Letrozole Effect on Metabolism, Bone Health, Oxidative Stress and Prostate in the Intact Adult Dog

**DOI:** 10.1002/vms3.70467

**Published:** 2025-06-25

**Authors:** Zahra Dadvand, Dorsa Zahedtalab, Maryam Barzegar Bafruei, Nooshin Derakhshandeh, Asghar Mogheiseh, Saeed Nazifi, Mohammad Reza Divar, Mahboobeh Ashrafi

**Affiliations:** ^1^ Department of Clinical Sciences School of Veterinary Medicine Shiraz University Shiraz Iran; ^2^ Department of Basic Sciences School of Veterinary Medicine Shiraz University Shiraz Iran

**Keywords:** aromatase inhibitors, dog, oxidative stress, prostate, vitamin D

## Abstract

Letrozole enhances male fertility by balancing oxidative stress and altering the hormonal regulation of spermatogenesis. The study aimed to determine the effect of short‐term letrozole administration on the concentration of canine prostatic‐specific esterase, parathormone, insulin‐like growth factor, vitamin D, alkaline phosphatase, lactate dehydrogenase, creatinine kinase, calcium, magnesium, cholesterol, and triglyceride in the serum of dogs. Moreover, total antioxidant capacity and lipid peroxidation were detected in intact adult dogs^,^ serum, and seminal plasma. Seven intact adult male mixed‐breeds were given 0.25 mg/kg of letrozole orally daily for four weeks. Blood samples and sperm were collected on days 0 (pretreatment), 14 (treatment), 28 (treatment), and 42 (post‐treatment). During the trial, malondialdehyde concentrations in serum and seminal plasma dropped considerably between day 0 and days 28 and 42 and between day 14 and days 28 and 42. The calcium concentration fell considerably on day 42 compared to days 0, 14 and 28. The reduction in 1,25‐dihydroxy vitamin D concentration was significantly different on days 14 and 42. The concentration of parathormone and alkaline phosphatase rose during and after treatment. However, the insulin‐like growth factor diminished during letrozole administration. According to the current research, oral administration of letrozole has been demonstrated to have a negative impact on bone health, leading to elevated levels of parathormone and alkaline phosphatase, as well as reduced levels of serum IGF‐1, calcium, and 1,25‐dihydroxy vitamin D. Serum levels of canine lipid markers, including cholesterol and triglycerides, and creatine kinase and lactate dehydrogenase levels were unaffected. The low number of dogs and the brief observation period limit the validity of the results and the inferences that can be drawn from the study.

## Graphical abstract

Letrozole increased the total antioxidant capacity and reduced malondialdehyde concentrations in both serum and seminal plasma. Letrozole increased parathormone and alkaline phosphatase levels in the serum. Letrozole decreased serum levels of IGF‐1, calcium, and 1, 25‐dihydroxy vitamin D.

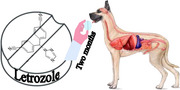

**Abbreviations**
ALP: alkaline phosphatase
BPH: Benign prostatic Hyperplasia
CK: creatine kinase
CPSE: canine prostatic specific esterase
DHT: dihydrotestosterone
IGF‐1: insulin‐like growth factor
LDH: lactate dehydrogenase
MDA: malondialdehyde
Mg: magnesium
RM ANOVA: Repeated measure analysis of variance
TAC: total antioxidant capacity

## 1 Background

The role of aromatase inhibitors in managing infertility, improving spermatogenesis, and the quality of semen in animals and humans has received increased attention across some studies in recent years. Letrozole, as an aromatase inhibitor, inhibits the conversion of androgens to oestrogens, which has a crucial impact on spermatogenesis according to the stimulation of LH and testosterone secretion (Barnadas et al., 2009).

A low testosterone concentration can raise oxidative stress, pro‐inflammatory cytokines, and interleukin levels in the testes (Vodo et al. 2013). Oxidative stress is one of the main reasons for male infertility. Khosravanian et al. (2014) showed that testosterone injection in rats with physical testicular injury diminished oxidative stress (Khosravanian et al. 2014). Consistent with the previous study, Cruz‐Topete et al. (2020) found that testosterone regulates the activity of antioxidant enzymes in adult rat cardiomyocytes and diminishes lipid peroxidation (Cruz‐Topete et al. 2020). Some clinical observations support this evidence that letrozole can support spermatogenesis by boosting endogenous testosterone levels (Gregoriou et al. 2012; Wang et al. 2018). Although raising the testosterone –to‐ oestrogen ratio improves the process, lowering the oestrogen level in other tissues (adipose tissue, musculoskeletal, and ovaries) might cause some complications. The oestrogen concentration considerably impacts osteoblasts, mature osteocytes, and lipoprotein metabolism (Rude, 1998). The main challenges patients face with aromatase inhibitors are skeletal muscle pain and a decline in bone mineral density. They have all been linked to low oestrogen levels, vitamin D deficiency, and insulin‐like growth factor 1 insufficiency (Waltman et al., 2009).

McCloskey et al. (2007) demonstrate a strong association between low oestrogen levels caused by the use of three kinds of aromatase inhibitors (letrozole, anastrozole, and exemestane) and bone resorption (McCloskey et al. 2007). Similarly, HDL levels and the HDL‐LDL ratio fell, most significantly in the exemestane group. Likewise, Goss et al. (2004) claim that letrozole's negative effects on ovariectomised rats include a drop in bone density and a change in lipid factors (Goss et al. 2004).

Clinical observations support that testosterone, dihydrotestosterone, and the balance of androgens and oestrogen drive prostate growth and maturation. The bioavailability of oestrogen in prostate tissue is decreased by blocking aromatase activity (Cheboub et al. 2019). Aromatase inhibitor‐treated mice can develop prostatic hypertrophy. Long‐term oestrogen deficiency may cause it (Shen, 2009; Cheboub et al. 2019). Hypoestrogenism leads to elevated blood cholesterol levels. Men with hypercholesterolaemia are at an increased risk of developing prostate cancer. Aromatase inhibitors raise cholesterol, which leads to amyloid deposition in the prostate lobes (McCloskey et al. 2007). So far, there have been very few studies about the effects of oral letrozole on lipids, oxidative stress, prostate gland function, and bone health in intact adult dogs. This research looks at the effects of letrozole on a variety of biological functions, including musculoskeletal markers (parathormone, insulin‐like growth factor, vitamin D, alkaline phosphatase, lactate dehydrogenase, creatine kinase, calcium, and magnesium), lipid profiles (triglyceride and cholesterol), canine prostatic specific esterase (a biomarker for prostatic function), oxidative stress, and lipid peroxidation in the serum and seminal plasma of intact adult dogs.

## 2 Material and Methods

### 2.1 Animals

Seven adult intact mixed‐breed male dogs weighing 20±2 kg and 2±1 years old were chosen. The dogs were fed 300 grams of commercial dog food per day (Nutri dry dog food; Behintash Co., Iran). The food analysis revealed at least 21% crude protein, 9% crude fat, 3% crude fibre, 0.8% salt, and 3000 Kcal/kg. They were adapted to the new condition for two weeks. Mebendazole (22 mg/kg; Parazol, ZagrosPharmed, Iran) and praziquantel (10 mg/kg; Lorensit, ZagrosPharmed, Iran) were used to treat dogs for parasites. Dogs were kept in separate pens and were fed dry dog food once a day and tap water ad libitum. Dogs were checked twice a day. In almost all visits, no food left over remained.

### 2.2 Study Design

Day zero was considered as a control time before oral administration of letrozole for 28 days and compared with other days of sampling. Blood and semen samples were drawn on days 0 (pretreatment), 14, 28 (treatment), and 42 (post‐treatment). For four weeks, the dogs received 0.25 mg/kg of oral letrozole tab (Letrofem 2.5 mg, Iran Hormone, Iran). The canine dose of letrozole was calculated using the following formula: [canine allometric coefficient (1.8) ×human dose (mg/kg)]. Blood samples were obtained from the jugular vein and placed in standard glass tubes. The semen samples were collected by manual masturbation into pre‐warmed (37°C) plastic tubes (Falcon, USA). The serum samples were centrifuged for 10 min at 3000 rpm. Serum and seminal samples were stored at ‐20°C.

### 2.3 Assay for Total Cholesterol, Triglycerides, Calcium, Magnesium, ALP, LDH, and CPK

Total cholesterol, triglycerides, calcium, magnesium, ALP, LDH, and CPK were measured with commercial kits (Pars Azmoon Co., Tehran, Iran) and were analysed using a biochemical autoanalyzer (Alpha Classic AT++, Sanjesh Company, Iran).

### 2.4 Assay for 25‐Hydroxy Vitamin D3 (25HVD3)

Serum 25‐hydroxy vitamin D3 (25HVD3) were measured by a quantitative sandwich enzyme immunoassay using a commercial dog‐specific competitive ELISA kit (CUSABIO, Shanghai, China, CSB‐E18032c). The sensitivity of the kit was <5 µg/L. The detection range of the kit was 20 µg/L‐100 µg/L. The intra‐assay precision and inter‐assay precision of the 25 ‐hydroxy vitamin D3 (25HVD3) kit were CV< 10% and CV< 15%, respectively.

### 2.5 Assay for Parathyroid Hormone (PTH)

PTH was measured by a quantitative sandwich enzyme immunoassay using a commercial dog‐specific competitive ELISA kit (CUSABIO, Shanghai, China, CSB‐E17575c). The sensitivity of the kit was 3.9 pg/mL. The detection range of the kit was 15.6 pg/mL‐1000 pg/mL. The intra‐assay precision and inter‐assay precision of the PTH kit were CV< 8% and CV< 10%, respectively.

### 2.6 Assay for Insulin Growth Factor 1(IGF‐1)

Serum IGF1 concentration was measured by a quantitative sandwich enzyme immunoassay using a commercial dog‐specific competitive ELISA kit (CUSABIO, Shanghai, China, CSB‐EL011086DO). The sensitivity of the kit was 7.8 ng/mL. The detection range of the kit was 31.25 ng/mL‐2000 ng/mL. The intra‐assay precision and inter‐assay precision of the IGF1 kit were CV< 8% and CV< 10%, respectively.

### 2.7 Assay for Canine Prostatic Specific Esterase (CPSE)

CPSE was measured by a quantitative enzyme‐linked immunosorbent assay (ELISA) type immunoassay on microwell using a commercial dog‐specific kit (Odelis CPSE, Virbac BVT, France). The sensitivity of the CPSE kit was 0.39 ng/ml. The intra‐assay precision and inter‐assay precision of the CPSE kit were CV<3.1% and CV<6.6%, respectively.

### 2.8 Assay for Total Antioxidant Capacity (TAC) and Malondialdehyde (MDA)

A commercial kit (ZellBio GmbH kit, Germany) determined the TAC level. The colour product of the chromogenic substrate (tetramethyl benzidine) emerged at the ending phase. The colour difference was calculated calorimetrically using a spectrophotometer (Jenway 6300 Spectrophotometer, UK) at 450 nm and represented as mmol/L. This method can determine TAC with 0.1 mM sensitivity (100 µmol/L). The intra‐ and inter‐assay CVs were below 3.4% and 4.2%, respectively. An assay kit from ZellBio GmbH (Germany) measured MDA (µmol/L; Cat. no. ZB‐MDA96A). In this kit, MDA is measured based on its reaction with thiobarbituric acid in an acidic condition and high temperature. The colour complex was measured colourimetrically at 535 nm. The assay kit sensitivity was 0.1 µM (inter‐assay CV: 5.8%) for MDA.

### 2.9 Statistical Analysis

The quantitative variables' mean and standard deviation were calculated using SPSS version 26 and reported. The one‐way repeated measures ANOVA and PostHoc tests were used to compare data collected on different days. The significance level was set at p < 0.05.

## 3 Results

### 3.1 Total Antioxidant Capacity Serum Concentration (TAC)

The serum concentration of TAC reached a maximum of 7.61±2.40 µmol/L after 28 days of treatment, up from the baseline of 3.96 ± 2.46 µmol/L (pre‐treatment or control). The serum concentration of TAC increased by 92.17% during the study. A significant increase in TAC concentration was observed on day 28 compared with day 0 (p = 0.04) and day 14 (p = 0.02; Table 1).

**TABLE 1:** The effect of letrozole administration (0.25 mg/kg, PO) on concentration (means±SEM) and changes of TAC and MDA in serum and semen samples studied before (day 0), during (days 14 and 28) and after (day 42) treatment of adult mixed‐breed dogs. Data were analysed using one‐way repeated‐measures analysis of variance (ANOVA) and Tukey's multiple comparison tests.

	Day				
Factors	0	14	28	42	Changes (%)
**The analysis of antioxidant potential**					
**TAC (µmol/l)**					
*Serum*	3.96±2.46^a^	3.58±2.76^a^	7.61±2.40^b^	7.15±2.01^ab^	92.17
*Seminal plasma*	7.85±2.59	7.58±2.86	10.70±2.42	10.47±2.59	33.37
**MDA (µmol/l)**					
*Serum*	0.56±0.03^a^	0.54±0.03^a^	0.44±0.04^b^	0.44±0.04^b^	−21.42
*Seminal plasma*	0.06±0.007^a^	0.06±0.004^a^	0.04±0.003^b^	0.04±0.002^b^	−33.33

*Notes*: Different letters in superscripts indicate statistically significant differences (p≤0.05).

Abbreviations: MDA = malondialdehyde, TAC = total antioxidant capacity.

### 3.2 Malondialdehyde Serum Concentration (MDA)

The serum MDA concentration decreased by 27% between day 0 (0.56±0.04 mol/L) compared with day 42(0.44±0.04 mol/L). The decreasing concentration of MDA between day 0 vs. days 28 and 42 (p < 0.0008) and between day 14 vs. days 28 and 42 (p = 0.0002) were significant (Table 1).

### 3.3 Total Antioxidant Capacity of Seminal Plasma (TAC)

Although results showed a rise in TAC concentrations of seminal plasma (48.61%) throughout the study, the differences were not found in TAC concentrations between various sampling days (Table 1).

### 3.4 Malondialdehyde Seminal Plasma Concentration (MDA)

MDA concentrations decreased from 0.060.007 mol/l at day 0 or control to 0.040.02 mol/l at day 42. The MDA concentrations between day 0 vs. days 28 and 42 (p < 0.05) and between day 14 vs. days 28 and 42 (p < 0.05) were significant and statistically significant (Table 1).

### 3.5 Canine Prostatic Specific Esterase Serum Concentration (CPSE)

CPSE concentration was increased from70.187 ±13.57 ng/ml (day 0, pre‐treatment) to a maximum concentration of 92.614±13.98 ng/ml on day 42. The concentration of serum CPSE increased by 31.94% during the study. No differences were found in canine prostatic‐specific esterase concentrations between sampling days (Table 2).

**TABLE 2:** The effect of letrozole administration (0.25 mg/kg, PO) on concentration (means±SEM) and changes of the musculoskeletal factors (Vitamin D, Calcium, ALP, IGF‐1, PTH, Mg, LDH, and CPK), lipid factors (Cholesterol and Triglyceride), and the hyperplasia of the prostate gland factor (CPSE) in serum samples studied before (day 0), during (days 14 and 28), and after (day 42) treatment of adult mixed‐breed dogs. Data were analysed using one‐way repeated‐measures analysis of variance (ANOVA) and Tukey's multiple comparison tests.

	Day				
Factors	0	14	28	42	Changes (%)
**The musculoskeletal factors**					
*Vitamin D(pg/ml)*	21±3.393	21.29±3.467^a^	20.77±3.451	18.57±3.097^b^	−11.57
*Calcium(mg/dl)*	9.994±0.332^a^	10.035±0.3417^c^	9.965±0.337^d^	9±0.374^b^	−9.90
*ALP(U/L)*	103.742±24.919	104.957±25.549	105.428±25.283	116.414±25.686	12.21
*IGF‐1(nmol/l)*	30.428±16.277	30.285±15.37	29.142±15.563	34.428±17.868	13.14
*PTH(pmol/l)*	6.714±2.883	6.628±2.964	6.657±3.345	9.7±3.212	44.56
*Mg*	1.965±0.328	1.98±0.33	1.961±0.384	1.764±0.388	−10.20
*LDH(U/L)*	103.428±34.98	104.142±35.291	105±35.194	104.571±38.086	1.11
*CPK(U/L)*	146.714±68.876	146.428±70.483	150±70.92	128.714±46.02	−12.26
**Lipid factors**					
*Cholesterol(mg/dl)*	156.785±9.675	154.642±9.735	157.728±12.742	155.442±13.217	−0.85
*Triglyceride(mg/dl)*	31.242±4.529	32.385±4.374	31.528±5.156	31.288±5.984	0.12
**The hyperplasia of the prostate gland factor**					
*CPSE(ng/ml)*	70.187±13.577	74.850±13.704	85.972±13.656	92.614±13.988	31.96

*Notes*: Different letters in superscripts indicate statistically significant differences (p ≤ 0.05).

Abbreviations: ALP = alkaline phosphatase, CPK = creatine kinase, CPSE = canine prostatic specific esterase, IGF‐1 = insulin‐like growth factor, LDH = lactate dehydrogenase, Mg = magnesium, PTH = parathormone.

### 3.6 1,25‐Dihydroxy Vitamin D Concentration

Concentrations of 1,25‐dihydroxy vitamin D dropped significantly between days 14 and 42, from 213.467 pg/ml to 183.097 pg/ml (P<0.05). 1,25‐dihydroxy vitamin D concentrations were not different on other days. 1,25‐dihydroxy vitamin D levels decreased by 11.5% throughout the study (Table 2).

### 3.7 Alkaline Phosphatase Concentration (ALP)

A rise in Alkaline phosphatase concentration (12%) was noted throughout the study; however, the differences were not found in ALP concentrations between the various sampling days (Table 2).

### 3.8 Parathyroid Hormone Concentration ()

The highest amount of parathormone hormone was associated with day 42, and a comparison of day 0 (pre‐treatment) with days 14 and 28 confirmed a steady decrease. No differences were found in concentrations between various days (Table 2).

### 3.9 Insulin Growth Factor 1 (IGF‐1)

The highest level of IGF‐1 was observed on day 42, and a comparison of day 0 (pre‐treatment) with days 14 and 28 demonstrated a steady decline. There were no differences in IGF‐1 concentrations between different sampling days (Table 2).

### 3.10 Serum Calcium Concentration (Ca)

The serum calcium concentration decreased from 9.994 ±0.332 mg/dl (day 0, pre‐treatment) to 9±0.374 mg/dl (day 42). Ca levels decreased by 10% during the study, but there was a slight variation. Ca concentrations on day 42 were significantly different from those on day 0 (p = 0.01), day 14 (p = 0.02), and day 28 (p = 0.01) (Table 2).

### 3.11 Serum Magnesium Concentration (Mg)

The minimum level of Mg was observed on day 42. There were no differences in Mg concentrations between different sampling days (Table 2).

### 3.12 Serum Lactate Dehydrogenase Concentration (LDH)

The highest LDH concentration was observed on day 28. Compared to day 0, the control group, days 14 and 28 show an increasing trend, while days 28 through 42 show a decreasing trend. There were no differences in LDH concentrations between different sampling days (Table 2).

### 3.13 Serum Creatine Kinase Concentration (CPK)

The highest concentration of CPK was observed on day 28, and when compared to the control group (day 0), days 14 and 28 demonstrate an increasing trend, whereas days 28 through 42 demonstrate a decreasing trend. There were no differences in CPK concentrations between various sampling days (Table 2).

### 3.14 Serum Cholesterol Concentration

The highest cholesterol level was on day 28, and there was no consistent increasing or decreasing trend when comparing the control group to other days of the study. There were no differences in cholesterol concentrations between various sampling days (Table 2).

### 3.15 Serum Triglyceride Concentration

The highest amount of triglycerides was associated with day 14, and a comparison of day 0 (pre‐treatment) with day 14 reveals an increasing trend. In contrast, comparing days 14 to 42 reveals a decreasing trend. There were no variations in triglyceride levels between different sampling days (Table 2).

## 4 Discussion

Letrozole, a third‐generation aromatase inhibitor, enhances reproductive performance by altering the concentration of hormones affecting spermatogenesis (increasing serum testosterone concentration and decreasing oestradiol serum level) and reducing oxidative stress. Testosterone is crucial for maintaining an oxidative stress equilibrium. In our study, oral letrozole (0.25 mg/kg) administration for 30 days improved total antioxidant capacity and decreased malondialdehyde concentration in male dogs.

Previous articles have mentioned how low testosterone concentration can raise reactive oxygen radicals (ROS) levels and pro‐inflammatory cytokines and interleukins in the testicular environment (Vodo et al. 2013; Khosravanian et al. 2014; De Ronde and de Jong, 2011; Jackson and Jackson, 1984; Kooshesh et al. 2020). Cruz‐Tepete et al. (2020) indicate that testosterone mitigates age‐related heart oxidative stress. Testosterone controls the expression and activity of the antioxidant enzymes (SOD and GSH‐Px) in adult rat cardiomyocytes by converting them to 17‐estradiol, thereby reducing lipid peroxidation (Cruz‐Topete et al. 2020). Since the aromatase enzyme is essential for oestrogen synthesis and converts androgens to oestradiol, aromatase inhibitors can stimulate LH production and lead to elevated blood and intratesticular testosterone levels (Santen, 1981; Pavlovich et al. 2001; Raman and Schlegel, 2002). Researchers found that methotrexate increased MDA, TNF‐, and IL‐1 concentrations and decreased SOD and GPx levels in rat testes. By working together, letrozole and methotrexate mitigate methotrexate's side effects, and letrozole's antioxidant and anti‐inflammatory properties offer testes protection analogous to testosterone propionate (Makary et al. 2018; Geisler, 2011; Furman et al. 2014; Malkin et al. 2004; Soljancic et al.; 2013; Xu et al. 2008)

As an aromatase inhibitor, letrozole prevents the conversion of testosterone to oestrogen in peripheral tissues by inhibiting the cytochrome P450 enzyme. This decreases the availability of oestrogen in various organs, such as fat tissue, skeletal muscle, bone, and ovaries. Consequently, it results in the occurrence of side effects (Rude, 1998). Letrozole side effects were studied in rats by Goss et al. (2004). Letrozole treatment for five months resulted in a decrease in bone density and changes in blood serum calcium. Joint and muscle pain, stiffness, and muscle weakness are common symptoms of musculoskeletal problems attributed to letrozole‐induced decrease in oestrogen levels and vitamin D deficiency (Goss et al. 2004; Elliott et al., 2020). Porosity and bone damage are inversely proportional to the serum oestrogen level; a low oestrogen level can harm the bones by causing the body to lose calcium, which is crucial to bone density (Waltman et al. 2009). Other studies have focused on the level of hydroxyvitamin D and its deficiency in breast cancer patients who received letrozole. The results indicated a decrease in 25‐hydroxyvitamin D and increased parathormone hormone and alkaline phosphatase concentration. In addition, because vitamin D aids in the absorption of calcium and phosphorus, a low serum concentration of 1,25‐dihydroxy vitamin D decreases serum calcium and magnesium concentrations. In addition, the low serum calcium concentration increases the secretion of parathyroid hormone, which ultimately stimulates vitamin D production (Vani et al. 2016). Consistent with the other literature on day 42 following letrozole administration in the present study, a rise in parathormone hormone was noted, in addition to a decreasing trend in serum calcium and 1,25 dihydroxy vitamin D concentrations on days 28 and 42 (Pilutin et al. 2021; Saki et al. 2019). In 2010, researchers set out to investigate the source of the musculoskeletal pain experienced by those taking letrozole by measuring their levels of 25‐hydroxyvitamin D deficiency. Vitamin D deficiency is known to be a side effect of letrozole treatment, so a 50,000‐unit vitamin D supplement was recommended every few months. The findings suggest that vitamin D supplementation may mitigate the side effects of letrozole (Khan et al. 2010). Yonden et al. (2009) determined whether or not the aromatase inhibitor letrozole affected the incidence of femur fracture and the concentrations of ALP, calcium, and phosphate in the serum of female rats (Yonden et al. 2009). While calcium and net bone area decreased, biomechanical values (ALP, phosphate) increased significantly with letrozole. These findings suggest that letrozole affects bone biomarkers like ALP, calcium, and phosphate in both intact and ovariectomized rats, suggesting that it may increase the risk of bone fracture. According to the findings of this study, ALP levels rise above the threshold in dogs treated with daily doses of 0.25 mg/kg letrozole for 4 weeks.

Although previous research has linked letrozole to pain and muscle weakness in those with vitamin D deficiency and the release of intracellular enzymes like CPK and LDH (Geisler, 2011), we found no evidence of this in the current study. The short period of letrozole administration and the low dose may account for the lack of significant change in the activity of the creatine kinase and lactate dehydrogenase enzymes in the serum of the tested dogs.

Although previous research demonstrated an inverse relationship between oestrogen levels and insulin‐like growth factor, with oestrogen‐treated women exhibiting lower levels of insulin‐like growth factor, Ca and vitamin D sufficiency are necessary for IGF‐1 production (Gallicchio et al. 2013; Cigler et al. 2010; Ferrari et al. 2002). Gallicchio et al. (2013) showed that aromatase inhibitors (AIs), the hormonal treatment for postmenopausal patients with oestrogen receptor‐positive breast cancer, frequently cause musculoskeletal pain (Gallicchio et al. 2013). Musculoskeletal pain in breast cancer patients has been significantly associated with decreases in IGF‐1 and vitamin D concentrations over the first six months of AI treatment. In the dogs of our research, letrozole did not significantly affect insulin‐like growth factors.

Magnesium is essential for bone strength and synthesizing organic bone matrix. However, the result of this study is similar to that of Pilutin et al. (2020) show that magnesium serum concentration does not vary significantly across experiment days (Pilutin et al. 2021).

It is also worth mentioning that oestrogen has other effects on the body. Several enzymes involved in the metabolism of high‐density lipoproteins and triglycerides are influenced by oestrogen production. Low‐density lipoprotein is broken down faster by oestrogen. Moreover, increasing HDL‐C concentration is crucial in enhancing the cardiovascular system (Mumford et al. 2010). Analysis of 341 papers that might be relevant to letrozole at a dose of 2.5 milligrams per day showed a substantial decrease in total cholesterol and HDL‐C in the letrozole group compared to the placebo group. Despite this, neither LDL‐C nor triglycerides changed (Palmisano et al. 2017; Manson and Bassuk, 2015; Ariadi et al. 2019; Ghaffari et al. 2018). In 2007, researchers examined the impact of letrozole, anastrozole, and exemestane, three aromatase inhibitor medications, on lipid variables in ninety postmenopausal women with normal bone density. Lipid variables changed in the exemestane group after 24 weeks (lower HDL and HDL‐LDL ratio), while there was no change in blood lipid content in the letrozole group, which is consistent with the results of our study (McCloskey et al. 2007). The brief duration of letrozole treatment may account for the absence of change in the examined dogs^,^ serum cholesterol, and triglyceride concentrations.

Aromatase inhibitors limit nitric oxide synthase activity by decreasing oestradiol production, leading to significantly lower nitric oxide levels. In mammals, nitric oxide plays a vital role in regulating prostate function. The symptoms of prostatic hyperplasia are caused by an increase in the tone and expansion of smooth muscle cells, which are promoted by a low amount of nitric oxide (Straub, 2007).

In adults, hyperphosphatemia can cause damage to the prostate, and oestrogen is essential for protecting against this. Researchers demonstrated that prostatic hyperplasia is caused by using aromatase inhibitors to decrease oestrogen, leading to hyperphosphatemia and lowering dihydroxy vitamin D levels (Cheboub et al. 2019; Straub, 2007; Cheboub et al. 2019). In the current investigation, a 28‐day course of oral letrozole treatment resulted in a significant elevation in CPSE concentrations (specific androgen‐dependent protein in canine prostate) in the serum.

## 5 Conclusion

**FIGURE 1:**
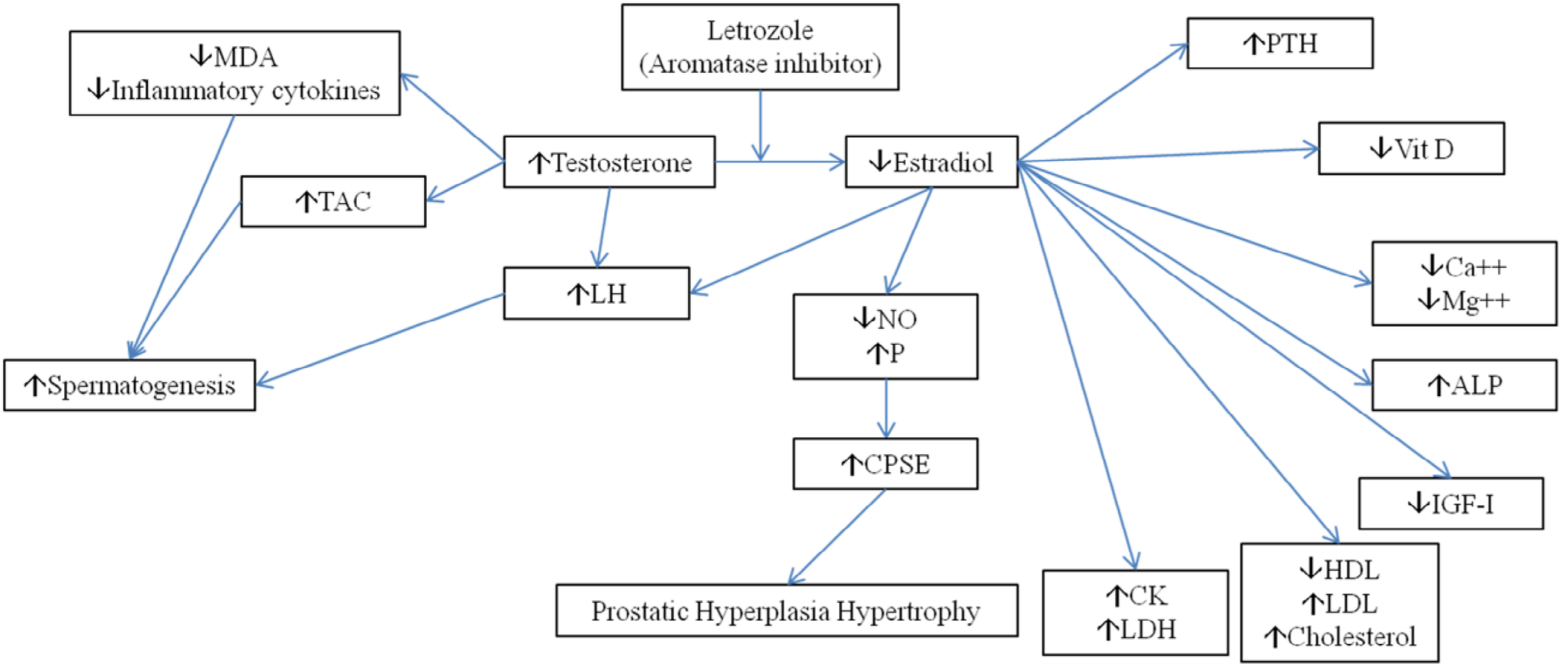
Schematic view of letrozole effects and related mechanisms on spermatogenesis, prostate function, bone health, and lipid metabolism. MDA: malondialdehyde, TAC: total antioxidant capacity, PTH: parathormone, ALP: alkaline phosphatase, IGF‐I: insulin‐like growth factor‐I, LDL: low‐density lipoprotein, HDL: high‐density lipoprotein, CK: creatine kinase, LDH: lactate dehydrogenase, CPSE: canine prostate‐specific esterase.

This study provides empirical evidence on the short‐term effects of letrozole, showing its potential to increase total antioxidant capacity and decrease malondialdehyde concentrations in serum and seminal plasma. The research highlights the impact of letrozole on lipid profile, prostate gland function, and bone health. Specifically, letrozole is found to have adverse effects on bone health, leading to elevated parathormone and alkaline phosphatase levels, alongside decreased serum IGF‐1, calcium, and 1,25‐dihydroxy vitamin D levels (refer to Figure 1). While levels of canine lipid markers, including cholesterol and triglycerides, as well as creatine kinase and lactate dehydrogenase levels, remained unchanged, the study notes limitations due to the small sample size and short observation period, influencing the robustness of the results and implications drawn from the study.

## Author Contributions

**Asghar Mogheiseh**: conceptualisation, methodology, investigation, data curation, writing – original draft, writing – review and editing. **Nooshin Derakhshandeh**: conceptualisation, methodology, investigation, data curation, writing – review and editing. **Saeed Nazifi**: study design, laboratory analysis, methodology, writing – review and editing. **Mohammad Reza Divar**: investigation, data collection, data analysis, writing – review and editing. **Dorsa Zahedtalab**: study design, laboratory analysis, writing – review and editing. **Zahra Dadvand**: data analysis, visualisation, writing – review and editing. **Mahboobeh Ashrafi**: laboratory analysis, methodology, writing – review and editing. **Maryam Barzegar Bafruei**: laboratory analysis, investigation, writing – review and editing

## Conflicts of Interest

The authors declare no conflict of interests.

## Declarations

Ethics approval and consent to participate

## Animal Rights Statement

This study was conducted under the supervision of the Society for the Iranian Prevention of Animal Cruelty and the Research Council of Shiraz University (IACUC No. 6387/63). The Framework for Animal Ethics carried out the experimental protocols. After the conclusion of the study, all dogs were castrated.

## Peer Review

The peer review history for this article is available at https://www.webofscience.com/api/gateway/wos/peer‐review/10.1002/vms3.70467.

## Data Availability

The datasets used and/or analysed during the current study are available from the corresponding author on reasonable request.
